# Decreased Incidence of Pediatric Intussusception during COVID-19

**DOI:** 10.3390/children8111072

**Published:** 2021-11-21

**Authors:** Jun Sung Park, Young-Hoon Byun, Seung Jun Choi, Jong Seung Lee, Jeong-Min Ryu, Jeong-Yong Lee

**Affiliations:** 1 Department of Pediatrics, Asan Medical Center, University of Ulsan College of Medicine, Seoul 05505, Korea; mrpiglet@naver.com (J.S.P.); sjchoi@catholic.ac.kr (S.J.C.); 2 Department of Emergency Medicine, CHA Bundang Medical Center, CHA University School of Medicine, Seongnam 13496, Korea; byunyoun@naver.com; 3 Department of Emergency Medicine, Asan Medical Center, University of Ulsan College of Medicine, Seoul 05505, Korea; jarvisyi@hanmail.net (J.S.L.); qweynaver@naver.com (J.-M.R.)

**Keywords:** intussusception, COVID-19, epidemiology, communicable disease

## Abstract

Coronavirus disease 2019 (COVID-19) changed the epidemiology of various diseases. The present study retrospectively investigates the epidemiologic and clinical changes in pediatric intussusception for ages ≤ 7 years before (February 2019–January 2020) and after (February 2020–January 2021) the COVID-19 outbreak in a single pediatric emergency department of a university-affiliated tertiary hospital. The incidence of communicable diseases—defined as infectious diseases with the potential for human-to-human transmission via all methods, non-communicable diseases, and intussusception were decreased following the COVID-19 outbreak (15,932 to 3880 (24.4%), 12,994 to 8050 (62.0%), and 87 to 27 (31.0%), respectively). The incidence of intussusception correlated significantly with the change in incidence of communicable diseases (Poisson log-linear regression, odds ratio = 2.15, 95% CI = 1.08–4.26, and *p* = 0.029). Compared with the pre-pandemic period, patients of the pandemic period showed higher proportions of pathologic leading point (PLP) and hospitalization (14.8% vs. 2.3% and 18.5% vs. 4.6%, respectively), lower base excesses (−4.8 mmol/L vs. −3.6 mmol/L), and higher lactate concentrations (1.7 mmol/L vs. 1.5 mmol/L). The incidence of pediatric intussusception decreased after the COVID-19 pandemic. This reduced incidence may be related to the reduced incidence of communicable diseases. However, the proportions of more severe diseases and PLPs were higher after the COVID-19 pandemic.

## 1. Introduction

Intussusception, defined as the invagination of one segment of the intestine within a more distal segment [1], is the most frequent cause of intestinal obstruction in children aged 5 months to 3 years and the most common abdominal surgery in children younger than 2 years [2]. The etiology of intussusception remains poorly understood, with structural lead points such as lymphoproliferative diseases, duplication cyst, etc., seen in only 3% of patients, whereas 60–100% have idiopathic intussusception [1,3]. Several infectious factors, such as rotavirus vaccination, non-enteric type adenovirus, and human herpes virus 6, have been reported to contribute to the pathogenesis of intussusception [4,5,6]. In addition, seasonal variations in intussusception suggest that infectious or climatic conditions contribute to its pathogenesis [4,6,7,8]. Epidemiological similarities, such as seasonality, may provide clues to the links between surgical conditions and communicable diseases [9,10].

Coronavirus disease 2019 (COVID-19) is an acute respiratory disease caused by severe acute respiratory syndrome coronavirus 2 [11]. It was declared a worldwide pandemic in March 2020, causing more than 100 million confirmed cases and more than 2 million deaths from then until January 2021 [12]. As more than 400,000 confirmed cases and 10,000 deaths are reported per day, many countries have adopted quarantine strategies, such as wearing masks, social distancing, and lockdowns, to reduce person-to-person contact and to prevent further spread [13]. In the past, such responses during the influenza H1N1 and Middle Eastern respiratory syndrome epidemics were found to reduce the incidence of other communicable diseases [14,15]. In Taiwan, these strategies during the COVID-19 pandemic resulted in incidental reductions in infectious diseases, such as influenza and pneumococcal diseases [16]. Moreover, the COVID-19 pandemic and its resultant quarantine strategies may have reduced the incidence of intussusception in pediatric patients [17]. In particular, South Korea responded to the pandemic situation using strong social lockdown measures and policies regarding the mandatory use of face masks, which lead to rapid control of COVID-19 transmissions [18]. Therefore, conducting a study on epidemiologic changes in diseases caused by social lockdowns is suitable.

The COVID-19 pandemic resulted in patient reluctance to visit the emergency department (ED) [19,20], which can delay various surgical emergencies and can worsen their clinical courses [21,22,23]. Especially in intussusception, the most common surgical emergency in young children, a delay in visiting the ED and in intervention can increase the risks of surgical reduction and poor outcomes [24,25,26,27]. Therefore, this study assessed the changes in the incidence and clinical characteristics of intussusception between the pre-pandemic and COVID-19 pandemic periods.

## 2. Materials and Methods

### 2.1. Study Design and Setting

This study retrospectively evaluated patients aged ≤ 7 years who were diagnosed with intussusception at the pediatric ED of a university-affiliated tertiary hospital between February 2019 and January 2021. Patients with a history of abdominal surgery, alleged intra-abdominal anomaly, and other medical conditions that have a risk of developing intussusception, such as Peutz–Jeghers syndrome and Henoch–Schönlein purpura, were excluded from this study. In order to evaluate the effect of dramatically decreased communicable diseases during the COVID-19 pandemic, we only included patients aged ≤ 7 years who were highly affected by the epidemiology of communicable diseases and had a higher proportion of idiopathic intussusception. Pediatric specialists at our research institute’s pediatric ED are available 24 h a day, and about 40,000 patients visit the hospital annually. The medical records showing the detailed demographic and clinical characteristics of the patients, including age, gender, medical history, laboratory results, and hospital course, were reviewed. All patients clinically suspected of having intussusceptions and who showed prolonged vomiting, cyclic irritability, currant jelly stool, palpable abdominal mass, and/or abdominal pain underwent sequential point-of-care ultrasound (POCUS), performed by ED physicians, and radiologist-performed ultrasound (RADUS), which includes evaluations of reducibility and PLP. Patients who underwent successful air enema reduction for the intussusception were discharged approximately 6 h later and monitored within the ED for complications and recurrence. By contrast, patients who experienced failures of more than two attempts at enema reduction were considered candidates for surgery. Beginning in January 2020, every patient visiting the ED who had symptoms of fever, cough, sputum, rhinorrhea, nasal stuffiness, myalgia, sore throat, headache, anosmia, dyspnea, and/or pneumonia within two weeks before the ED visit or had an epidemiologic association with COVID-19 exposure underwent COVID-19 testing by real-time reverse transcription polymerase chain reaction [28]. Clinical evaluations of intussusception in the ED were not altered during the study period, regardless of the COVID-19 outbreak. A recurrence of intussusception within 48 h was considered the same case, whereas a recurrence after 48 h was considered a separate case. This study was approved by the Institutional Review Board for Human Research of Asan Medical Center (IRB No. 2021-0082).

To analyze the epidemiologic changes in ED visits, information on 39,818 patients under age 7 years who visited the same ED during the same study period, 12 months, was collected. None were excluded. The basic demographic characteristics and diagnoses of these patients were recorded. Diagnoses were coded according to the International Statistical Classification of Diseases and Related Health Problems, tenth Revision version 2019 (ICD-10 ver. 2019) by ED physicians who were specialists in pediatrics or emergency medicine and had experience using ICD-10 codes for at least 7 years. The diagnostic codes were classified into two groups: communicable and non-communicable diseases. Communicable diseases included all codes of groups A and B, corresponding to certain infectious and parasitic diseases, and codes of infectious diseases for each system (e.g., G03, meningitis in the nervous system; H66, acute otitis media in the ear and mastoid process; and J18, pneumonia in the respiratory system). To include all possible infectious diseases, the specific codes from group R, corresponding to the symptoms, signs, and abnormal clinical and laboratory findings not otherwise classified, were included: fever, cough, sputum, rhinorrhea, vomiting, and diarrhea. All diagnoses that did not correspond to these descriptions were classified as non-communicable diseases.

Although the pan11 March 2020, we defined the time point for dividing the pre-pandemic from the pandemic periods as 1 February 2020. The reason was an earlier outbreak of COVID-19 at the research institute (Figure S1) [29]. The pre-pandemic period was defined as the 1-year period from February 2019 to January 2020, and the pandemic period was defined as the 1-year period from February 2020 to January 2021. The local COVID-19 epidemiology and lockdown status of the research institution were determined from press releases by government institutions [30].

### 2.2. Measures and Outcomes

The primary outcome was the incidence of intussusception during the year before and the year after the outbreak of the COVID-19 pandemic. The secondary outcome was the correlation between epidemiological changes in communicable diseases and the incidence of intussusceptions. The last outcome was the differences in the clinical characteristics of patients with intussusception before and after the outbreak of COVID-19.

### 2.3. Data Analyses

Descriptive data on the COVID-19 outbreak and ED visits were recorded and expressed using a graph and table, as determined by Excel 365 (Microsoft Corp., Redmond, WA, USA). The continuous variables before and after the outbreak of the pandemic were compared using Mann–Whitney U tests after testing the normality of distribution using Kolmogorov–Smirnov tests, and the categorical variables were compared using the χ^2^ test or Fisher’s exact test, as appropriate. Correlations between the incidence of intussusception and disease categories were determined by Poisson log-linear regression analyses. All statistical analyses were performed using PASW Statistics for Windows Version 18.0 (SPSS Inc., Chicago, IL, USA), with *p* values < 0.05 considered statistically significant.

## 3. Results

### 3.1. Epidemiology of COVID-19 Outbreak at the Study Location

Epidemiological information on the COVID-19 outbreak at the local research institution is presented in Figure S1. Three outbreaks of COVID-19 occurred in 2020, in February, August, and December. Measures limiting the use of or locking down all public facilities, schools, and daycare centers were implemented immediately after the first outbreak and continued due to repeated outbreaks.

### 3.2. Incidence of Intussusception and Its Association with Monthly ED Visits

**Figure 1:**
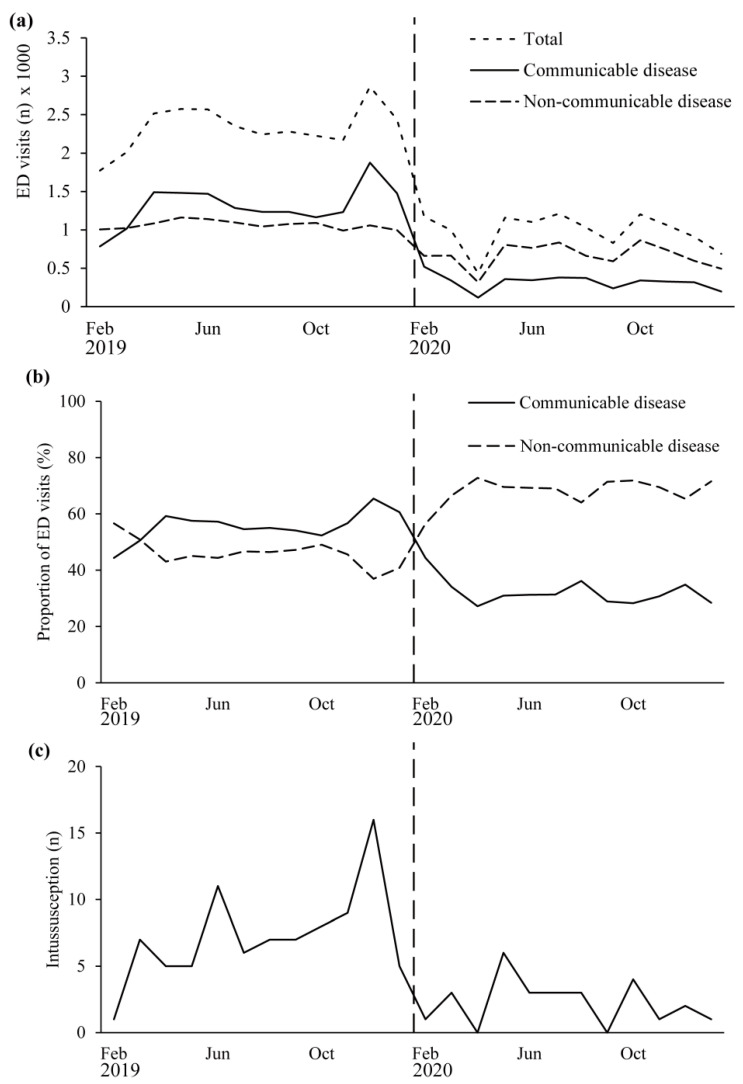
Monthly emergency department (ED) visits and number of children aged ≤7 years with communicable diseases, non-communicable diseases, and intussusception during the year before and the year after the outbreak of coronavirus disease 2019 (COVID-19). (**a**) Monthly ED visits during the overall study period. (**b**) Monthly proportion of communicable and non-communicable diseases over total ED visits. (**c**) Monthly number of patients with intussusception. Vertical dotted line: COVID-19 outbreak and social lockdown.

Figure 1 shows the monthly ED visits by patients aged ≤ 7 years during the 1-year pre-pandemic period from February 2019 to January 2020 and during the 1-year pandemic period from February 2020 to January 2021. In this age group, 28,018 patients visited the ED during the pre-pandemic period, but only 11,800 (42.1%) visited the ED after the outbreak (Table 1). ED visits for communicable diseases decreased from 15,912 to 3,880 (24.4%), and the proportions also decreased from 56.9% to 32.9% during the pandemic period compared with the pre-pandemic period. On the other hand, although visits for non-communicable diseases decreased from 12,994 to 5080 (62%), the proportions increased from 46.4% to 68.2% during the pandemic period compared with the pre-pandemic period.

During the 2-year study period, 114 patients were diagnosed with intussusceptions, but none were diagnosed with COVID-19. Of these 114 patients, 87 were diagnosed with intussusception during the pre-pandemic year, whereas 27 were diagnosed after the outbreak of COVID-19, which is a 69.0% reduction. A Poisson log-linear regression analysis showed that only monthly visits for communicable diseases significantly correlated with the incidence of intussusception (odds ratio = 1.000764, 95% CI = 1.000077–1.001451, and *p* = 0.029; Figure S2).

### 3.3. Clinical Characteristics of Intussusception before and during the COVID-19 Pandemic

Table 2 summarizes the basic demographic and clinical characteristics of pediatric patients diagnosed with intussusception in the pre-pandemic and pandemic periods. Age at onset, sex, length of ED stay, previous history of intussusception, rate of rotavirus vaccination, concomitant gastrointestinal tract infection, symptoms of upper respiratory tract infection, duration of illness, and initial presentations did not differ between the two periods (all *p* ≥ 0.05). The clinical course of patients with intussusception is summarized in Table 2. Time until air enema reduction was significantly longer during the pandemic period than during the pre-pandemic period (3.1 h vs. 2.6 h, *p* < 0.001), but no differences were found in the initial (*p* = 0.09) and final (*p* = 0.14) rates of successful reduction. The recurrence rates did not differ significantly, but hospital admission rate (18.5% vs. 4.6%, *p* = 0.03) and PLP frequency (14.8% vs. 2.3%, *p* = 0.03) were higher during the pandemic than during the pre-pandemic period. Laboratory testing showed that base excess (BE) was lower (−4.8 mml/L vs. −3.6 mmol/L, *p* = 0.03) and that serum lactate concentration was higher (1.7 mmol/L vs. 1.5 mmol/L, *p* = 0.02) after compared to before the outbreak of COVID-19.

Results are presented as number (%) or median (interquartile range).

## 4. Discussion

The present study showed that the incidences of both ED visits and intussusception were lower during the pandemic period compared with the pre-pandemic period of COVID-19. The decrease in ED visits for communicable diseases was more pronounced than the decrease in visits for non-communicable diseases, with the former significantly correlating with the incidence of intussusception. This result may suggest that communicable diseases participate in the pathogenesis of intussusception. In addition, intussusceptions following the COVID-19 outbreak had a higher proportion of PLPs and showed more deteriorated statuses on laboratory tests than intussusceptions before the pandemic.

Epidemiological studies have attempted to determine the correlation between intussusception and communicable diseases [7,8,31,32]. As these studies are oriented toward seasonal or epidemiological changes in gastroenteritis, the variables can confound each other. To our knowledge, the present study is the first to report that a marked reduction in the incidence of communicable diseases correlated with a reduction in the incidence of intussusception during the same time 12-month period for the same season and climate. As the incidence of intussusception due to PLP increased between the two study periods, the change in the incidence of intussusception is mainly due to a reduction in communicable diseases. The decrease in ED visits for intussusception indicates a decrease in its actual incidence because intussusception is hard to reduce spontaneously and requires immediate reduction; otherwise, it can be fatal [25]. However, although the dramatic reduction in ED visits for communicable diseases observed after the COVID-19 outbreak is consistent with global trends, changes in ED visits for communicable diseases may not reflect actual changes in their incidence [16,20,33,34]. As with other surgical emergencies, patients with relatively mild symptoms could be reluctant to visit the ED and their numbers may be underestimated due to fear of contact with COVID-19 and limited medical resources [19,21,22,23].

Mesenteric lymphoid tissue swelling or bowel wall thickening may partially explain the relationship between non-specific communicable diseases and intussusception [5]. Enteric viruses, including astroviruses, enteroviruses, noroviruses, and caliciviruses, are frequently asymptomatic or are undetected but may be a trigger or predisposing factor for the development of intussusception [35]. In addition, COVID-19-associated intussusception may result from the widespread immune activation occurring in COVID-19 [36,37,38,39]. Although none of the patients in this study were confirmed as having COVID-19, non-specific communicable diseases other than gastrointestinal tract infection might activate the immune system, resulting in hypertrophy of intestinal Peyer’s patches and inducing intussusception. However, changes in physical activity and the administration of antibiotics after the COVID-19 outbreak, factors not considered in this study, can act as confounders [40,41].

Although the basic demographic and clinical characteristics did not differ in patients who visited the ED before and after the outbreak of COVID-19, PLPs were more frequent during the pandemic period. Compared with idiopathic intussusception, intussusceptions due to PLP have been associated with poorer outcomes and may not be amenable to standard treatment owing to differences in their intussusception locations [27,42]. Indeed, differences were found in the serum lactate levels and base excess, which are possible markers of organ failure, in patients assessed before and after the COVID-19 outbreak. In particular, high serum lactate levels have been associated with poor outcomes of intussusception [26]. Although air enema reduction was delayed for about 0.5 h in patients who visited during the pandemic period, the duration of illness in these patients was similar to that in patients who visited during the pre-pandemic period. Patients underwent laboratory tests immediately after POCUS and fluid resuscitation, indicating that organ failure had already progressed rather than that treatment was delayed. The differences in patient characteristics before and after the outbreak of COVID-19 indicate that patients with intussusception during the COVID-19 outbreak may require more careful efforts to identify the PLP and to undergo rapid resuscitation. However, because our study included a relatively small number of patients, with small numerical differences between groups, studies in larger patient populations are needed to confirm the clinical significance of our findings.

This study had several limitations. First, because this study was retrospective, based on previously recorded ICD-10 codes, some patients had an R code for their primary complaint rather than a concrete diagnosis. Inter-rater variation could not be completely controlled, ED chart reviews are flawed by nature [43], and not all of the patients underwent diagnostic tests to identify specific pathogens. In addition, due to the inability of retrospective analyses of ICD-10 codes to accurately describe the affected system or specific pathogen, the correlations between communicable diseases and the incidence of intussusception were likely flawed. Second, the statistical power of this study was likely reduced due to the small number of patients with intussusception. Third, it is important to understand the epidemiological peculiarities of the South Korea population, which is smaller than those of countries in Europe and the USA, and of more sufficient medical infrastructure than developing countries. Furthermore, this study only assessed a single center and should be interpreted with much more caution for applications and generalizations of the study results. Therefore, prospective large-scale, multi-center studies that include follow-up with individual patients are required to overcome these limitations.

## 5. Conclusions

The incidence of pediatric intussusception after the COVID-19 pandemic began was lower than before the COVID-19 pandemic. This reduced incidence may be related to the reduced incidence of communicable diseases. However, the proportion of more serious diseases and of PLPs after the COVID-19 outbreak was higher than before the COVID-19 pandemic.

## Figures and Tables

**Table 1:** Annual emergency department (ED) visits before and after the COVID-19 outbreak.

	Pre-Pandemic Period	Pandemic Period
	Feb. 2019 to Jan. 2020	Feb. 2020 to Jan. 2021
Total visits	28,018	11,800 (42.1% ^a^)
Communicable disease	15,932	3880 (24.4% ^a^)
proportion ^b^, %	56.9	32.9 (57.8% ^a^)
Non-communicable disease	12,994	8050 (62.0%)
proportion ^b^, %	46.4	68.2 (147% ^a^)
Intussusception	87	27 (31% ^a^)
COVID-19 confirmed	0	0 (0% ^a^)

Results are presented as number or %. ^a^ Relative to the pre-pandemic period. ^b^ Relative to the total visits during each period.

**Table 2:** Clinical characteristics of patients with intussusception before and after the COVID-19 outbreak.

Variables		Non-Pandemic	Pandemic	Total	*p* Value
		*n* = 87	*n* = 27	*n* = 114	
Age, month		25 (16–37)	24 (14–31)	24.5 (16–36)	0.582
Sex, male		63 (72.4)	23 (85.2)	86 (75.4)	0.210
Past history of intussusception		9 (10.3)	6 (22.2)	15 (13.2)	0.188
Rotavirus vaccination		78 (89.7)	24 (88.9)	102 (89.5)	1.000
Concomitant GITI		21 (24.1)	6 (22.2)	27 (23.7)	1.000
Concomitant URTI		13 (14.9)	4 (14.8)	17 (14.9)	1.000
Duration of illness		13 (4–48)	9 (4–24)	12 (4–24)	0.140
Symptoms					
	Abdominal pain	83 (95.4)	25 (92.6)	108 (94.7)	0.626
	Cyclic irritability	74 (85.1)	22 (81.5)	96 (84.2)	0.763
	Vomit	31 (35.6)	11 (40.7)	42 (36.8)	0.653
	Hematochezia	14 (16.1)	8 (29.6)	22 (19.3)	0.161
EDLOS, h		9.5 (7.9–11.4)	9.2 (7.7–11.5)	9.4 (7.8–11.4)	0.886
ED course					
	Time to diagnosis	0.6 (0.3–1)	0.8 (0.4–1.6)	0.6 (0.4–1.1)	0.063
	Time to air reduction	2.6 (1.8–3.6)	3.1 (2.5–4.5)	2.9 (2.0–3.7)	0.000
	Success to air reduction	86 (98.9)	25 (92.6)	111 (97.4)	0.139
Admission		4 (4.6)	5 (18.5)	9 (7.9)	0.033
Pathologic leading point		2 (2.3)	4 (14.8)	6 (5.3)	0.027
Laboratory test					
	WBC, ×10^3^/µL	10 (7.6–12.9)	10.5 (8–15.6)	10.3 (7.9–13.2)	0.437
	CRP, mg/dL	0.2 (0.1–0.6)	0.3 (0.1–0.7)	0.2 (0.1–0.6)	0.839
	Bicarbonate, mmol/L	19.6 (18.4–21.1)	19.2 (17.7–20.9)	19.6 (18.3–21.1)	0.454
	BE, mmol/L	−3.6 (−4.9–−2.3)	−4.8 (−5.5–−3.7)	−4 (−5.1–−2.6)	0.030
	lactate, mmol/L	1.5 (1.1–1.8)	1.7 (1.4–2.4)	1.5 (1.2–1.9)	0.016
COVID-19 confirmed		0 (0)	0 (0)	0 (0)	NA

Abbreviations: COVID-19, coronavirus disease 2019; GITI, gastrointestinal tract infection; URTI, upper respiratory tract infection; EDLOS, length of ED stay; ED, emergency department; WBC, white blood cell; CRP, c-reactive protein; BE, base excess; NA, not applicable.

## Data Availability

The datasets generated and analyzed during the current study are not publicly available because the IRB of the Asan Medical Center (IRB numbers: 2021-0082) does not allow data to be shared with out-of-hospital facilities due to ethical consideration. However, the datasets are available upon request.

## Supplementary Materials

The following are available online at https://www.mdpi.com/article/10.3390/children8111072/s1, Figure S1: Incidence of COVID-19 for the location of the research institute, Figure S2: Poisson log-linear regression of monthly ED visits for the incidence of intussusception.

## References

[1] World Health Organization Acute Intussusception in Infants and Children: Incidence, Clinical Presentation and Management: A Global Perspective. https://apps.who.int/iris/handle/10665/67720.

[2] Kliegman R.M., Geme J.W.S., Blum N.J., Shah S.S., Tasker R.C., Wilson K.M. (2020). Ileus, adhesions, intussusception, and closed-loop obstructions. Nelson Textbook of Pediatrics.

[3] Huppertz H.-I., Soriano-Gabarró M., Grimprel E., Franco E., Mezner Z., Desselberger U., Smit Y., Bosch J.W.-V.D., De Vos B., Giaquinto C. (2006). Intussusception Among Young Children in Europe. Pediatr. Infect. Dis. J..

[4] Kim J.-S., Lee S.K., Ko D.-H., Hyun J., Kim H.-S., Song W., Kim H.S. (2017). Associations of Adenovirus Genotypes in Korean Acute Gastroenteritis Patients with Respiratory Symptoms and Intussusception. BioMed Res. Int..

[5] Kombo L.A., Gerber M.A., Pickering L.K., Atreya C.D., Breiman R.F. (2001). Intussusception, infection, and immunization: Summary of a workshop on rotavirus. PEDIATRICS.

[6] Hsu H.-Y., Kao C.-L., Huang L.-M., NI Y.-H., Lai H.-S., Lin F.-Y., Chang M.-H. (1998). Viral etiology of intussusception in Taiwanese childhood. Pediatr. Infect. Dis. J..

[7] Jo S., Lim I.S., Chae S.A., Yun S.W., Lee N.M., Kim S.Y., Yi D.Y. (2019). Characteristics of intussusception among children in Korea: A nationwide epidemiological study. BMC Pediatr..

[8] Guo W.-L., Zhang S.-F., Li J.-E., Wang J. (2014). Association of Meteorological Factors with Pediatric Intussusception in Subtropical China: A 5-Year Analysis. PLoS ONE.

[9] Stein G.Y., Rath-Wolfson L., Zeidman A., Atar E., Marcus O., Joubran S., Ram E. (2012). Sex differences in the epidemiology, seasonal variation, and trends in the management of patients with acute appendicitis. Langenbeck’s Arch. Surg..

[10] Khan M.S., Shahzad N., Arshad S., Shariff A.H. (2019). Seasonal Variation in Acute Cholecystitis: An Analysis of Cholecystectomies Spanning Three Decades. J. Surg. Res..

[11] Guan W.-J., Ni Z.-Y., Hu Y., Liang W.-H., Ou C.-Q., He J.-X., Liu L., Shan H., Lei C.-L., Hui D.S.C. (2020). Clinical Characteristics of Coronavirus Disease 2019 in China. N. Engl. J. Med..

[12] World Health Statistics 2020: A Visual Summary. https://www.who.int/data/gho/whs-2020-visual-summary.

[13] Zhang K., Vilches T.N., Tariq M., Galvani A.P., Moghadas S.M. (2020). The impact of mask-wearing and shelter-in-place on COVID-19 outbreaks in the United States. Int. J. Infect. Dis..

[14] Lee S.Y., Khang Y.-H., Lim H.-K. (2019). Impact of the 2015 Middle East Respiratory Syndrome Outbreak on Emergency Care Utilization and Mortality in South Korea. Yonsei Med. J..

[15] Schanzer D.L., Schwartz B. (2013). Impact of Seasonal and Pandemic Influenza on Emergency Department Visits, 2003–2010, Ontario, Canada. Acad. Emerg. Med..

[16] Galvin C.J., Li Y.-C., Malwade S., Syed-Abdul S. (2020). COVID-19 preventive measures showing an unintended decline in infectious diseases in Taiwan. Int. J. Infect. Dis..

[17] Zheng J., Ye Y., Liao Y., Bin Wang P. (2020). Fewer paediatric intussusception cases during the COVID-19 pandemic. J. Paediatr. Child. Health.

[18] Lee D., Heo K., Seo Y. (2020). COVID-19 in South Korea: Lessons for developing countries. World Dev..

[19] Lazzerini M., Barbi E., Apicella A., Marchetti F., Cardinale F., Trobia G. (2020). Delayed access or provision of care in Italy resulting from fear of COVID-19. Lancet Child. Adolesc. Health.

[20] Dopfer C., Wetzke M., Scharff A.Z., Mueller F., Dressler F., Baumann U., Sasse M., Hansen G., Jablonka A., Happle C. (2020). COVID-19 related reduction in pediatric emergency healthcare utilization—A concerning trend. BMC Pediatr..

[21] Lazzati A., Rousseau M.R., Bartier S., Dabi Y., Challine A., Haddad B., Herta N., Souied E., Ortala M., Epaud S. (2021). Impact of COVID-19 on surgical emergencies: Nationwide analysis. BJS Open.

[22] Burgard M., Cherbanyk F., Nassiopoulos K., Malekzadeh S., Pugin F., Egger B. (2021). An effect of the COVID-19 pandemic: Significantly more complicated appendicitis due to delayed presentation of patients!. PLoS ONE.

[23] Pogorelić Z., Milanović K., Veršić A.B., Pasini M., Divković D., Pavlović O., Lučev J., Žufić V. (2021). Is there an increased incidence of orchiectomy in pediatric patients with acute testicular torsion during COVID-19 pandemic?—A retrospective multicenter study. J. Pediatr. Urol..

[24] Chung J.L., Kong M.S., Lin J.N., Wang K.L., Lou C.C., Wong H.F. (1994). Intussusception in infants and children: Risk factors leading to surgical reduction. J. Formos. Med. Assoc..

[25] Lampl B.S., Glaab J., Ayyala R.S., Kanchi R., Ruzal-Shapiro C.B. (2017). Is Intussusception a Middle-of-the-Night Emergency?. Pediatr. Emerg. Care.

[26] Lee J.-Y., Byun Y.-H., Park J.-S., Lee J.S., Ryu J.-M., Choi S.J. (2020). Lactic acid level as an outcome predictor in pediatric patients with intussusception in the emergency department. BMC Pediatr..

[27] Loukas M., Pellerin M., Kimball Z., de la Garza-Jordan J., Tubbs R.S., Jordan R. (2011). Intussusception: An anatomical perspective with review of the literature. Clin. Anat..

[28] Hong K.H., Lee S.W., Kim T.S., Huh H.J., Lee J., Kim S.Y., Park J.-S., Kim G.J., Sung H., Roh K.H. (2020). Guidelines for Laboratory Diagnosis of Coronavirus Disease 2019 (COVID-19) in Korea. Ann. Lab. Med..

[29] Choi J.Y. (2020). COVID-19 in South Korea. Postgrad. Med. J..

[30] Coronavirus Disease-19, Republic of Korea. http://ncov.mohw.go.kr/en.

[31] Chen S.C.-C., Wang J.-D., Hsu H.-Y., Leong M.-M., Tok T.-S., Chin Y.-Y. (2010). Epidemiology of Childhood Intussusception and Determinants of Recurrence and Operation: Analysis of National Health Insurance Data Between 1998 and 2007 in Taiwan. Pediatr. Neonatol..

[32] Jang J., Lee Y.J., Kim J.S., Chung J.-Y., Chang S., Lee K., Choe B.-H., Hong S.J., Song J.S., Park K.Y. (2017). Epidemiological Correlation between Fecal Adenovirus Subgroups and Pediatric Intussusception in Korea. J. Korean Med. Sci..

[33] Goldman R.D., Grafstein E., Barclay N., Irvine M.A., Portales-Casamar E. (2020). Paediatric patients seen in 18 emergency departments during the COVID-19 pandemic. Emerg. Med. J..

[34] Chong S.-L., Soo J.S.L., Allen J.C., Ganapathy S., Lee K.P., Tyebally A., Yung C.F., Thoon K.C., Ng Y.H., Oh J.Y. (2020). Impact of COVID-19 on pediatric emergencies and hospitalizations in Singapore. BMC Pediatr..

[35] Okimoto S., Hyodo S., Yamamoto M., Nakamura K., Kobayashi M. (2011). Association of viral isolates from stool samples with intussusception in children. Int. J. Infect. Dis..

[36] Bazuaye-Ekwuyasi E.A., Camacho A.C., Rios F.S., Torck A., Choi W.J., Aigbivbalu E.E., Mehdi M.Q., Shelton K.J., Radhakrishnan G.L., Radhakrishnan R.S. (2020). Intussusception in a child with COVID-19 in the USA. Emerg. Radiol..

[37] Moazzam Z., Salim A., Ashraf A., Jehan F., Arshad M. (2020). Intussusception in an infant as a manifestation of COVID-19. J. Pediatr. Surg. Case Rep..

[38] Cai X., Ma Y., Li S., Chen Y., Rong Z., Li W. (2020). Clinical Characteristics of 5 COVID-19 Cases With Non-respiratory Symptoms as the First Manifestation in Children. Front. Pediatr..

[39] Martínez-Castaño I., Calabuig-Barbero E., Gonzálvez-Piñera J., López-Ayala J.M. (2020). COVID-19 Infection Is a Diagnostic Challenge in Infants With Ileocecal Intussusception. Pediatr. Emerg. Care.

[40] Hviid A., Svanström H. (2009). Antibiotic use and intussusception in early childhood. J. Antimicrob. Chemother..

[41] Shahidi S.H., Williams J.S., Hassani F. (2020). Physical activity during COVID-19 quarantine. Acta Paediatr..

[42] Applegate K.E. (2009). Intussusception in children: Evidence-based diagnosis and treatment. Pediatr. Radiol..

[43] Gilbert E.H., Lowenstein S.R., Koziol-McLain J., Barta D.C., Steiner J. (1996). Chart Reviews in Emergency Medicine Research: Where Are the Methods?. Ann. Emerg. Med..

