# Supplementary material for: Decreased Incidence of Pediatric Intussusception during COVID-19
Source: Children (Basel). 2021 Nov 21;8(11):1072. doi: 10.3390/children8111072 (PMC8625463; doi:10.3390/children8111072)
Supplement: Supplementary file 1 [file children-08-01072-s001.zip › children-1429754-SI.pdf]

Figure S1. Incidence of COVID-19 in the location of the research institute

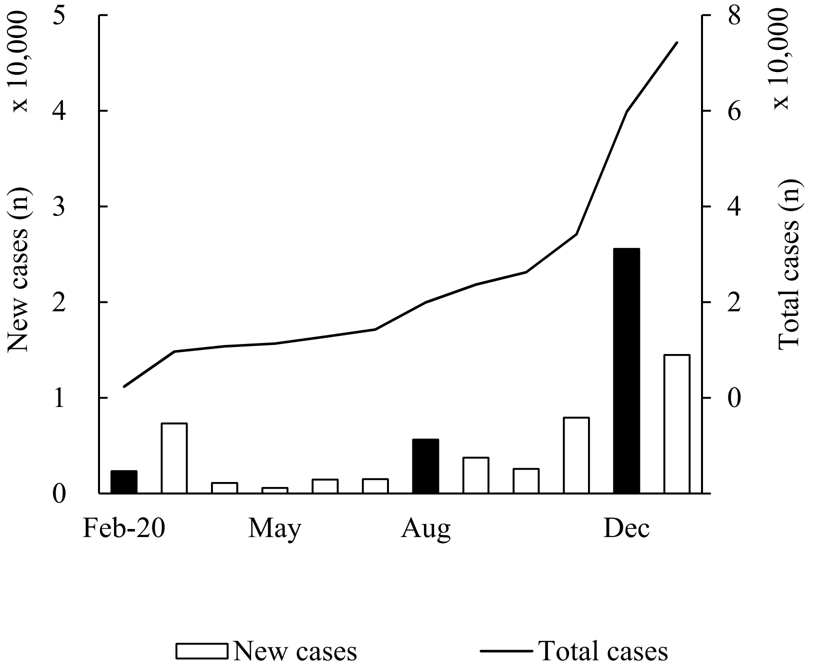

	New cases	Total cases
**Feb-20**	**2337**	**2337**
Mar-20	7324	9661
Apr-20	1100	10761
May-20	583	11344
Jun-20	1456	12800
Jul-20	1505	14305
**Aug-20**	**5642**	**19947**
Sep-20	3752	23699
Oct-20	2572	26271
Nov-20	7930	34201
**Dec-20**	**25572**	**59773**
Jan-21	14489	74262

Abbreviation: COVID-19, coronavirus disease 2019.
Bold numbers and black bars: outbreaks

Coronavirus Disease-19, Republic of Korea: Korea Disease Control and Prevention Agency; 2021 [Available from: http://ncov.mohw.go.kr/en]

Figure S2. Poisson log-linear regression of monthly ED visits toward incidence of intussusception.

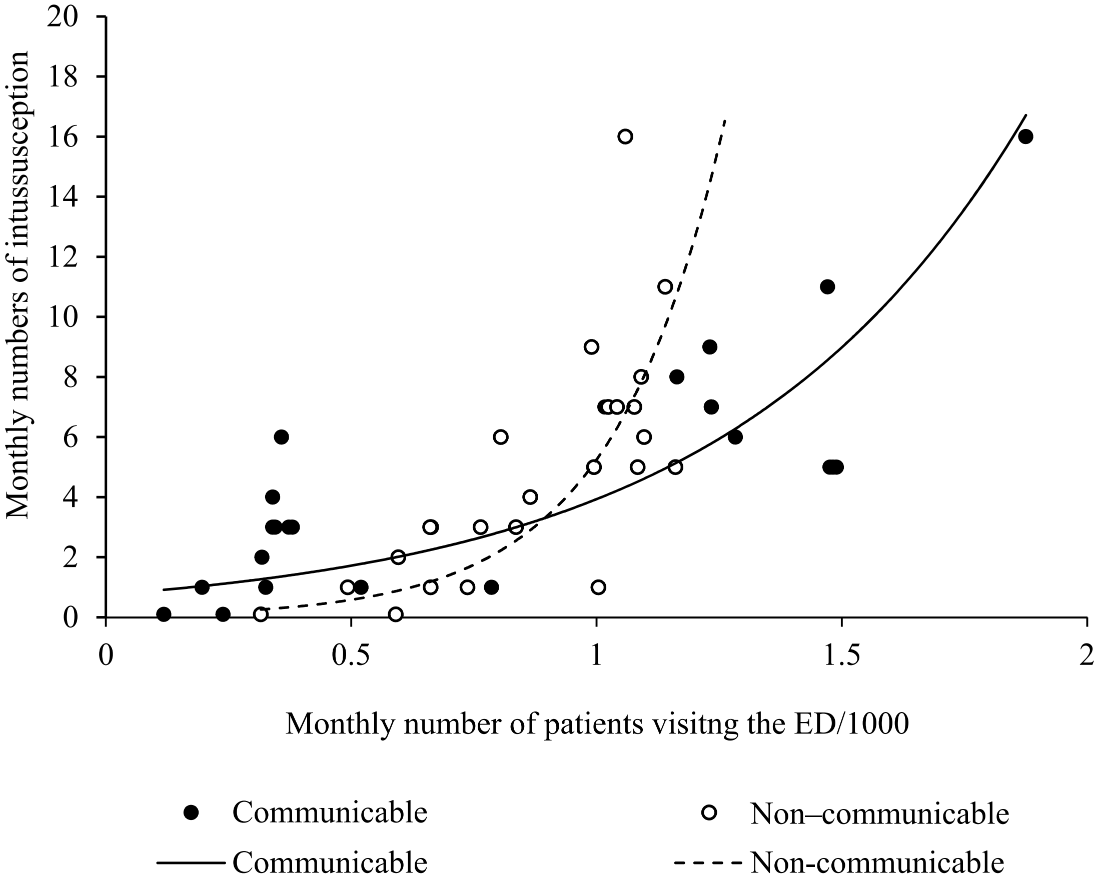

Variables	B	Odds ratio	95% CI	*P* value
Communicable disease/1,000	0.76	2.15	1.08–4.26	0.029
Non-communicable disease/1,000	1.39	4.00	0.57–28.02	0.163
